# Entropy of never born protein sequences

**DOI:** 10.1186/2193-1801-2-200

**Published:** 2013-04-30

**Authors:** Grzegorz Szoniec, Maciej J Ogorzalek

**Affiliations:** Information Technology Department, Faculty of Physics, Astronomy and Applied Computer Science, Jagiellonian University, 4 Reymonta Street, Cracow, 30-059 Poland

**Keywords:** Never born protein, Block entropy, Relative entropy, Kullback-Leibler divergence, Origin of life

## Abstract

**Background:**

A Never Born protein is a theoretical protein which does not occur in nature. The reason why some proteins were selected and some were not during evolution is not known. We applied information theory to find similarities and differences in information content in Never Born and natural proteins.

**Findings:**

Both block and relative entropies are similar what means that both protein kinds contain strongly random sequences.

An artificially generated Never Born protein sequence is closely as random as a natural one.

**Conclusions:**

Information theory approach suggests that protein selection during evolution was rather random/non-deterministic.

Natural proteins have no noticeable unique features in information theory sense.

**Electronic supplementary material:**

The online version of this article (doi:10.1186/2193-1801-2-200) contains supplementary material, which is available to authorized users.

## Introduction

Existing and known proteins are only a small subset of all possible sequences. Why were only some proteins selected during evolution? The reason is not known but two possible ways are considered: deterministic and random. To investigate theoretical sequences of amino acids a term Never Born Protein was introduced (Chiarabelli et al. 2006). Since 2006 only a few papers about them have been published. The most significant research has shown that 20% of them fold (i.e. reach stable and functional 3D structure) in laboratory conditions (Chiarabelli et al. 2006) and a tool for generating sequences with no similarity to natural proteins has been developed – Random Blast (Evangelista et al. 2007). The high folding ratio has been positively surprising and has abated opinion that existing proteins are the only stable and folding sequences. Surprisingly, as 1 out of 5 absolutely randomly generated proteins was a possibly useful one for living organisms. The authors did not expect so high percentage, furthermore their results came with doubts about correctness of their approach/methodology. Up to now this has been the most important discovery in Never Born protein science.

The question about proteins origin is still open. There are papers that proved natural and synthetic (random) proteins are not different (Jacob 1969; Luisi 2003) or only slightly different from each other(Weiss et al. 2000), there are also papers that proved these two groups of proteins are significantly different and protein selection during evolution was a driven process (Munteanu et al. 2008; De Lucrezia et al. 2012). The results depend on methodology (which is not discussed here because of limited scope of this report) nevertheless another open question is which approach should be considered as more correct and reliable than others.

Information theory (Shannon 1948) is applied to almost any branch of science. Information quantities i.e. Shannon entropy H (Shannon 1948) and Kullback–Leibler divergence (or relative entropy) D_KL_ (Kullback & Leibler 1951) are defined as

1 $$H\;=\;-\sum_{i}p_{i}\log\left(p_{i}\right)$$

2 $$D_{\mathrm{KL}}\left(P||Q\right)\;=\;\sum_{i}p_{i}\log\left(p_{i}/q_{i}\right)$$

Where P and Q are probability densities, P = {p_i_} and Q = {q_i_}. Shannon entropy is a measure of uncertainty in an outcome with probability p_i_ and relative entropy is a measure of similarity between two probability densities (it is not a true metric i.e. D_KL_(P||Q) is not equal to D_KL_(Q||P) except when P = Q).

In protein science, information properties of natural proteins were comprehensively studied (Strait & Dewey 1996) and further intensively developed e.g. (Dewey 1996). In this short report we apply some of those ideas to Never Born proteins and we show that protein picking during evolution is closer to be a random/non-deterministic process. Our approach is strictly theoretical as (Strait & Dewey 1996).

## Methods and results

**Figure 1:**
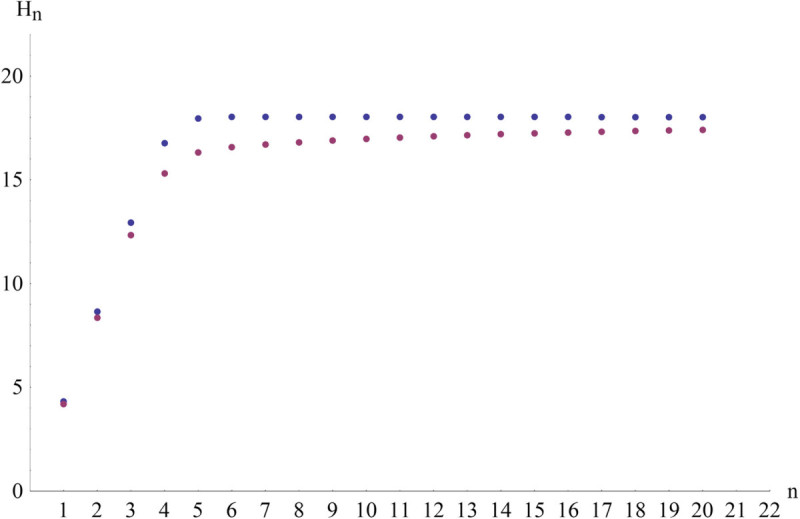
**Block entropies H**_**n**_ **(in bits) vs. block lengths n of never born (blue) and natural (purple) proteins.**

Natural proteins were randomly picked from UniProt database (The UniProt Consortium 2012). Never Born Proteins were generated with Random Blast tool (in both cases the number of sequences is 1250, in total around 400,000 amino acids, sequences lengths vary from 61 to around 1700 amino acids). Shannon entropies were calculated not only for every amino acid but also for blocks (block entropy (Papadimitriou et al. 2010)) of length from 2 to 20. Block entropy was calculated identically like Shannon entropy but probabilities refered to amino acid subsequences (blocks) of a specific length. Probabilities were normalized over occurrences in all sequences (the data and the scripts are available at http://www.cyfronet.pl/~myszonie/ent). The results are presented in Figure 1 and Table 1.

**Table 1:** **Relative entropies between never born and natural proteins**

D_KL_(P_NBP_||Q_NP_)	D_KL_(P_NP_||Q_NBP_)
0.1554	0.1307

## Conclusions

The plot (Figure 1) proves that the values of entropy for both groups of proteins are very close. It means that uncertainty or in other words a number of possible amino acid combinations, is almost the same. This indicates that natural protein sequences are random like Never Born Protein sequences. Moreover, relative entropy values show that encoding a natural protein sequence using probability density of Never Born Proteins requires only a small excess of information (and vice versa). Summing up the inferences, protein selection during evolution is – in an information theory approach - closer to be a random process than deterministic one what is in-line with (Jacob 1969; Jacob 2003; Weiss et al. 2000). There is still a doubt wheter that small difference does not play a key role.

## Authors’ original submitted files for images

Below are the links to the authors’ original submitted files for images. Authors’ original file for figure 1
