# Supplementary figures and images for: Entropy of never born protein sequences
Source: Springerplus. 2013 Apr 30;2(1):200. doi: 10.1186/2193-1801-2-200 (PMC3671101; doi:10.1186/2193-1801-2-200)

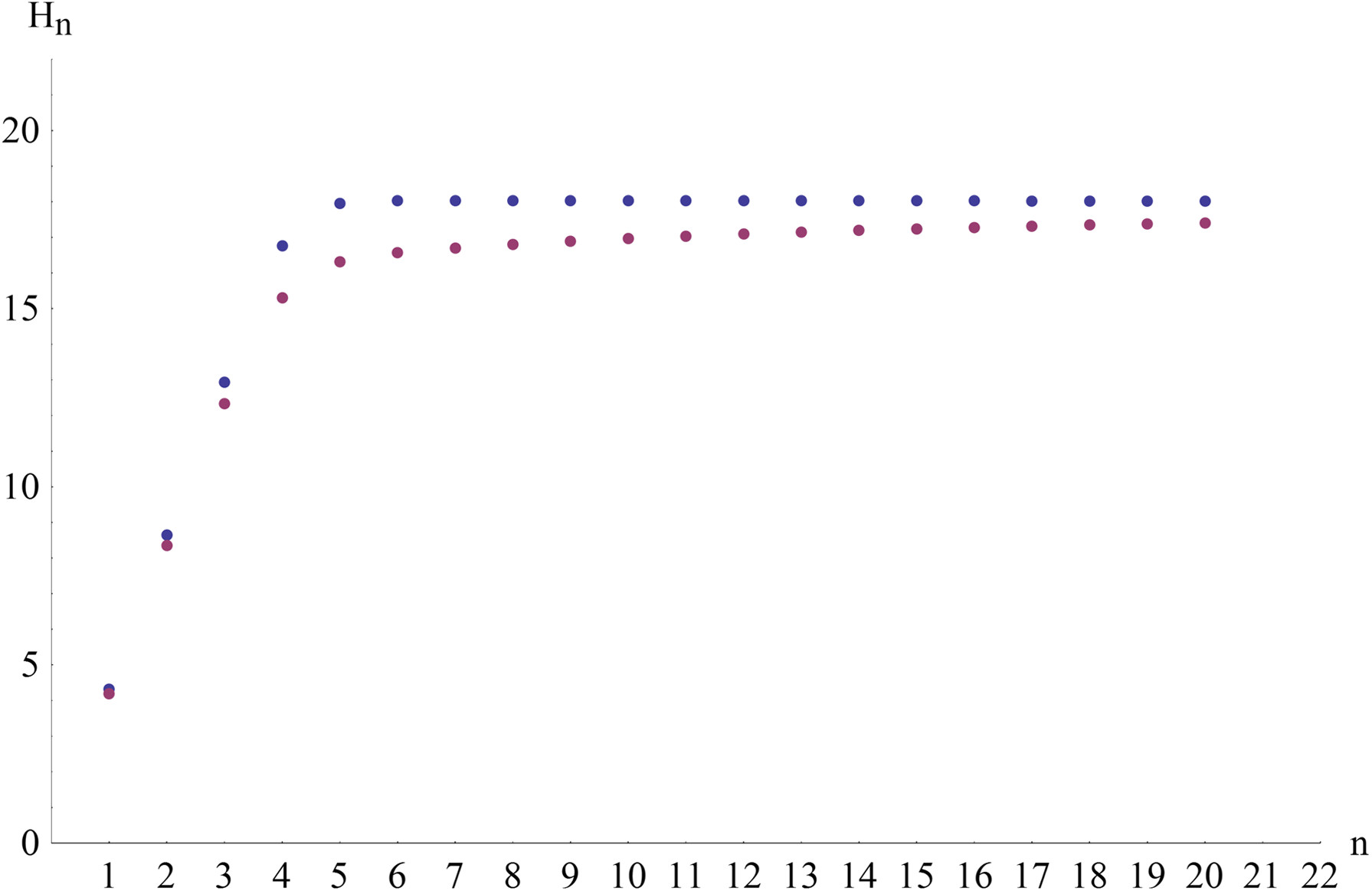

Supplement: Supplementary file 1 — Authors’ original file for figure 1 [file 40064_2012_301_MOESM1_ESM.jpeg]
